# Nanoparticle/Metal–Organic Framework Composites for Catalytic Applications: Current Status and Perspective

**DOI:** 10.3390/molecules22122103

**Published:** 2017-11-30

**Authors:** Wenlong Xiang, Yueping Zhang, Hongfei Lin, Chang-jun Liu

**Affiliations:** 1 Collaborative Innovation Center of Chemical Science and Engineering, School of Chemical Engineering and Technology, Tianjin University, Tianjin 300072, China; WenlongX@tju.edu.cn; 2 Department of Chemistry, Tianjin University, Tianjin 300350, China; ypzhang@tju.edu.cn; 3 Voiland School of Chemical Engineering and Bioengineering, Washington State University, Pullman, WA 99164, USA

**Keywords:** metal–organic frameworks, MOF, nanoparticle, composites, catalyst, photocatalyst

## Abstract

Nanoparticle/metal–organic frameworks (MOF) based composites have recently attracted significant attention as a new class of catalysts. Such composites possess the unique features of MOFs (including clearly defined crystal structure, high surface area, single site catalyst, special confined nanopore, tunable, and uniform pore structure), but avoid some intrinsic weaknesses (like limited electrical conductivity and lack in the “conventional” catalytically active sites). This review summarizes the developed strategies for the fabrication of nanoparticle/MOF composites for catalyst uses, including the strategy using MOFs as host materials to hold and stabilize the guest nanoparticles, the strategy with subsequent MOF growth/assembly around pre-synthesized nanoparticles and the strategy mixing the precursors of NPs and MOFs together, followed by self-assembly process or post-treatment or post-modification. The applications of nanoparticle/MOF composites for CO oxidation, CO_2_ conversion, hydrogen production, organic transformations, and degradation of pollutants have been discussed. Superior catalytic performances in these reactions have been demonstrated. Challenges and future developments are finally addressed.

## 1. Introduction

Metal–organic frameworks (MOFs) are a class of highly porous crystalline materials, which are assembled by the coordination of metal ions with organic ligands [1,2,3,4]. Due to the reversibly self-correcting kinetic characteristics that are controlled by the moderate coordination bond energies, MOF materials have exhibited their unique features, such as clearly defined crystal structures, high surface areas, tunable and uniform pore structures, and special confined nanopore microenvironments [5,6]. These features make them promising materials for numerous applications. Nevertheless, a few weaknesses, such as limited electrical conductivity, poor thermal, and chemical stability, prohibit MOFs from exhibiting their full potential for practical applications. Therefore, it is urgently needed to further enhance the functional properties of MOF materials. Recently, the attempts have been made by combining MOFs with various functional materials to produce new functional composites. These MOF based composite materials can integrate the excellent properties and mitigate the shortcomings of the individual components. The synergistic effects of both MOFs and other active components may cause the composites to possess new properties and unprecedented performance, which are not attainable by the individual parts. Up to now, MOF composites have been successfully fabricated with incorporating various active species, including metal or metal oxides nanoparticles (NPs), carbon materials, polymers, polyoxometalates (POMs), quantum dots (QDs), ionic liquids (ILs), and so on [7,8,9,10,11,12,13]. These MOF composites have been widely employed in the areas of gas storage, separation, sensing, biomedicine, protection of bio-macromolecules, and catalysis [14,15,16,17,18,19,20,21,22,23].

In term of catalytic application, MOF composites show some superior characteristics because of the unique features of MOFs mentioned above. For example, high porosity with ordered crystalline pores and high surface areas contribute to the uniform dispersion and the high density of catalytic sites, which can improve the catalytic efficiency [5]. The confined pore sizes can limit active species growth and agglomeration, and selectively transport different substrate molecules for size-selective catalysis [24,25]. These superior characteristics have made these composite materials promising in heterogeneous catalysis.

**Scheme 1:**
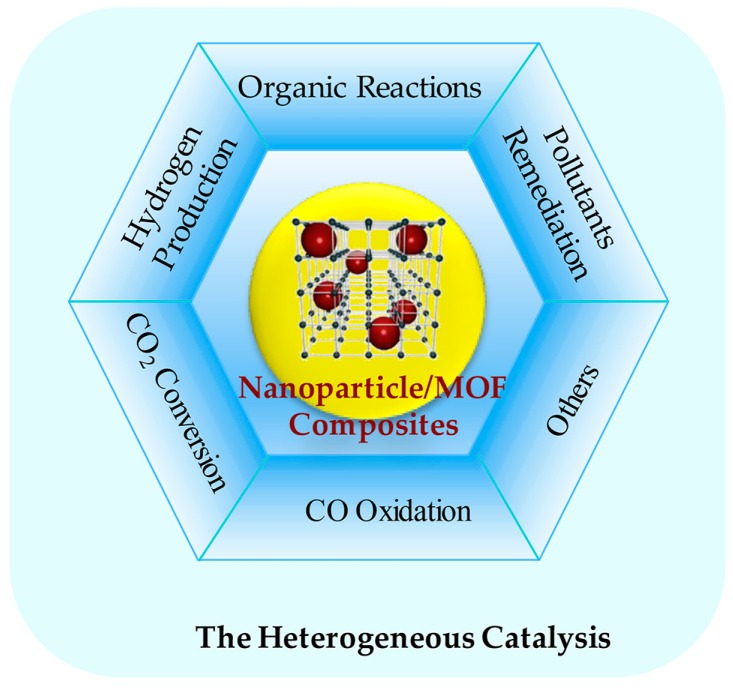
Heterogeneous catalysis of nanoparticle/metal–organic frameworks (MOF) composites.

To date, even though MOF composites, as heterogeneous catalysts, are still at a developing stage, a series of catalytic investigations have been reported. It has been demonstrated that MOF composites as heterogeneous catalysts have showed notable catalytic behavior, such as high activity, good stability, and reusability [5,8,12,16,17,18]. Although some reviews have been published covering some topics on heterogeneous catalysis of MOF composites, this review aims to summarize the recent advances in this hot topic, including CO oxidation, CO_2_ conversion, hydrogen production, organic reaction and pollutants remediation, and so on, over nanoparticle/MOF composite based catalysts (Scheme 1).

## 2. General Synthesis of Nanoparticle/MOF Composites

There are three main established approaches for the immobilization of functional molecules or nanoscale objects in MOFs, known as ‘‘ship in bottle’’ approach, ‘‘bottle around ship’’ approach, and one-step synthesis approach.

**Figure 1:**
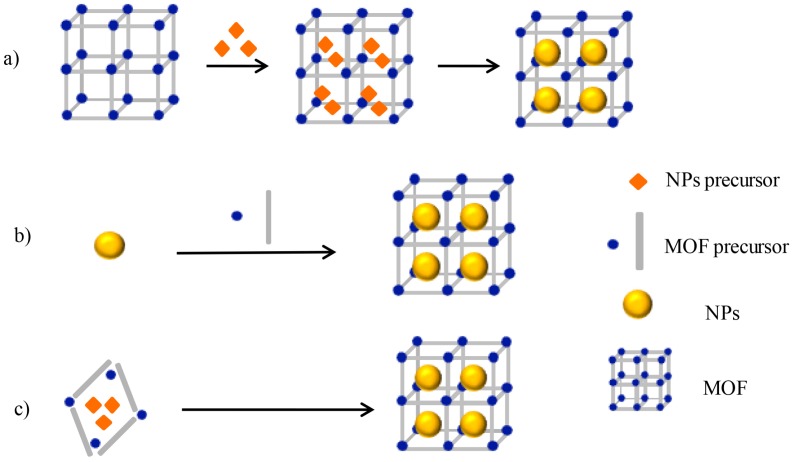
Main approaches for the fabrication of nanoparticle/MOF composites. (**a**) Ship in bottle; (**b**) bottle around ship; and, (**c**) in situ one-step synthesis.

The ‘‘ship in bottle’’ approach involves the encapsulation of active small molecules components or NPs precursors in the cavities of MOFs, followed by further treatment leading to the desired functional structure (Figure 1a). Various techniques, such as solution infiltration, vapor deposition, and solid grinding have been exploited for introducing the NPs precursors into MOFs [7,26]. It is significantly challenging to precisely control the location, composition, structure, and morphology of incorporated guests when using this synthesis strategy. Zhang et al. [27] summarized the recent progresses in the size and structure control of MOF supported noble metal catalysts. In addition, the possible formation of the precursors and products on the external surface of MOFs needs to be considered. In order to avoid NPs aggregation on the external surface of MOFs, a double-solvent method (DSM) was successfully developed to rationally introduce precursors into MOF pores, followed by further treatment, to produce NPs@MOF composites [9,28]. The DSM is based on a hydrophilic and hydrophobic solvent, and the large cages with hydrophilic environments and high pore volumes in some MOFs. The quantitative volume (less than the MOF pore volume) of the aqueous precursor solution can be readily incorporated into the pores of MOF, which was suspended in a large amount of low-boiling-point hydrophobic solvent, by capillary force and hydrophilic interactions. By using the DSM combined with the reduction with reducing agents, such as H_2_ and NaBH_4_, metallic NPs including Pt, Pd, Rh, Ni NPs, and AuNi, RuNi, AuCo, and CuCo bimetallic NPs were successfully immobilized inside the pores of MOFs without aggregation on the external surface of the framework [28,29,30,31,32,33].

The “bottle around ship” approach, also known as the template synthesis approach, generally involves two steps (Figure 1b). Firstly, the functional molecules or NPs are synthesized individually and often stabilized by capping agents or surfactants. Subsequently, the pre-synthesized nanoscale objects are added into a synthetic solution containing MOF precursors to assemble the MOF. The nanoscale objects do not occupy the pore space of the MOF, but instead are surrounded by grown MOF materials. By using this method, the problems of the aggregation of NPs on the external surface of MOFs are limited, the size, morphology, and structure of entrapped NPs can be easily controlled because they are preformed prior to the assembly of MOF framework [26,34]. However, the introduction of NPs sometimes results in difficulties with the subsequent growth of the MOF because of the high interfacial energy barrier between the two kinds of materials. In addition, the presence of the capping agents (for example, polyvinyl pyrrolidone PVP) might be unfavorable for the complete exposure of active sites, and even alter or degrade the performance of the NPs.

A one-step synthesis approach involves directly mixing the precursors solution of NPs and MOFs together, followed by the simultaneous growth of NPs and MOF, and assembling both of them into a nanostructure (Figure 1c). When compared with the above stepwise approaches, this strategy is straightforward and simple, but it usually needs to balance the rates of the self-nucleation and growth of the NPs and MOFs. In particular, the choice of the functional groups in organic linkers or solvents is vital for trapping the NPs precursors and stabilizing the NPs that were formed in situ and for facilitating the hetero-nucleation of the MOFs on the surface of the NPs [9,35].

The different preparation methods of the catalyst may result in a noticeable difference of catalytic performance. For example, the uniform Pt NPs were successfully supported on or encapsulated inside MOF particles to prepare Pt/UiO-66-NH_2_ and Pt@UiO-66-NH_2_ catalysts, through the solution infiltration technique (the ‘‘ship in bottle’’ approach) and “bottle around ship” approach, respectively [36]. The different methods cause a difference in the Pt location relative to the MOF, which leads to a very different photocatalytic activity. The internal Pt NPs in the MOF greatly shortens the path of electron transfer from MOF to Pt NPs than supported Pt. As a result, Pt@UiO-66-NH_2_ exhibits much better charge-carrier utilization, and thus significantly higher photocatalytic activity towards hydrogen production than Pt/UiO-66-NH_2_. Furthermore, Pt@UiO-66-NH_2_ possesses excellent stability and recyclability as a result of the great confinement for Pt NPs in the MOF.

## 3. Catalysis Applications

### 3.1. Catalytic CO Oxidation

CO oxidation has been extensively investigated in the field of heterogeneous catalysis due to the fundamental interest and its close relevance in practical applications, such as gas sensors for the detection of trace amounts of CO, automotive exhaust gas treatment, and polymer electrolyte fuel cells [37,38,39,40]. Herein, we addressed the CO oxidation reaction based on metal or metal oxide NPs/MOF composites. Table 1 summarizes the catalytic activities for CO oxidation of several MOF-supported NPs catalysts [28,41,42,43,44,45,46,47,48,49]. Generally, MOF-supported NPs catalysts have shown good catalytic performance for CO oxidation at relatively high temperatures.

Xu et al. [41] reported a pioneering study that described MOF-supported noble metal NPs as an efficient catalyst for CO oxidation. The catalytic activity over Au@ZIF-8 for CO oxidation increases with increasing Au loading from 0.5 to 5 wt %, accompanied by the decrease of the temperature of 50% conversion of CO from 225 °C to 170 °C. The 5 wt % Au@ZIF-8 achieves a complete conversion of CO at approximately 210 °C. Afterwards, it was reported that the total conversion of CO was achieved at around 200 °C by the Pt and Au NPs, supported on NH_2_-MIL-101(Al) and UIO-66, respectively [42,43]. Importantly, the reaction temperature of complete conversion of CO can be further reduced to below 150 °C by incorporating Pt, Pd, and Ag NPs with apposite MOF supports [28,44,45,46,47,48]. Wang and co-workers proposed that both the size of the metal NPs and the nature of the support play an important role on the catalytic performance of Pt@UIO-67 for CO oxidation through a combination study of experiment and DFT calculation [45]. EL-Shall et al. [46] reported high CO oxidation activities over Pd NPs supported on Ce-MOF. The Pd@Ce-MOF catalyst with 5 wt % Pd loading shows surprisingly high catalytic activity, with a complete conversion at 96 °C. The authors proposed that the high activity was mostly attributed to the interaction of the Pd NPs and the Ce sites within Ce-MOF.

In addition to MOF-supported noble metal NPs, MOF-supported metal oxide NPs as an active catalyst for CO oxidation was reported. Wang et al. [49] firstly employed ZIF-8 as host to prepare hexagonal Co_3_O_4_ NPs via the thermolysis of cobalt nitrate that is accommodated in the pores of the MOF host at a low temperature of 200 °C. The Co_3_O_4_@ZIF-8 composite exhibited excellent catalytic activity for CO oxidation, which was related to the highly dispersed Co_3_O_4_ NPs in the well-retained MOF networks. Complete conversion of CO was achieved at 80 °C by the resulting composite catalyst with good cycling stability and long-term stability. Furthermore, this synthesis method can be easily extended to the preparation of other metal oxide NPs.

### 3.2. Catalytic CO_2_ Conversion

Catalytic conversion of CO_2_ into valuable chemicals, such as CO, CH_4_, CH_3_OH, HCOOH, cyclic carbonates, and so on, has consistently drawn significant attention [16,18,50,51,52,53]. The MOF based composites have been developed as active catalysts for the conversion of CO_2_.

**Figure 2:**
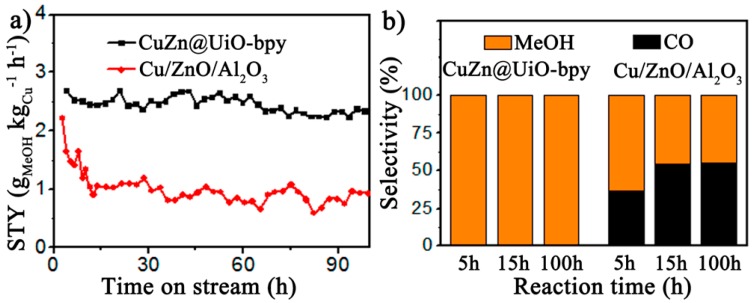
(**a**) The space-time yield (STY) of MeOH vs reaction time over a period of 100 h on stream; (**b**) Selectivity of product vs reaction time. Reproduced with permission from Reference [55]. Copyright 2017, American Chemical Society.

Recently, metal or metal oxide NPs incorporated in MOFs have been proved to be effective catalysts for converting CO_2_ to valuable chemicals, including CO, CH_4_, CH_3_OH, and light olefins [33,53,54,55,56,57]. The Materials of Institute Lavoisior (MIL) and University of Oslo (UiO) families, and their surface modified MOFs were mainly used as supports due to their high thermal stability and high chemical stability in water. An efficient catalyst, which was prepared by encapsulating single Cu nanocrystal (18 nm) into UiO-66, was reported recently for CO_2_ hydrogenation to methanol. It shows a steady eight-fold yield over the benchmark Cu/ZnO/Al_2_O_3_ catalyst, with a 100% selectivity to methanol [54]. Interestingly, Wang and co-workers recently extended this approach with the use of a UiO-bpy MOF, which anchored ultrafine Cu/ZnO*_x_* NPs within the pores to restrain the agglomeration of Cu NPs and phase separation between Cu and ZnO*_x_* [55]. As shown in Figure 2, the resulting Cu/ZnO*_x_*@MOF catalysts exhibit remarkably higher activity (space-time yield of 2.59 g_MeOH_ kg_Cu_^−1^ h^−1^), higher selectivity (100%), and higher stability (>100 h) for methanol synthesis from CO_2_ hydrogenation, when compared to the commercial Cu/ZnO/Al_2_O_3_ catalyst. Similarly, Lu et al. [33] prepared Ni NPs encapsulated in MIL-101(Cr) composites by double solvent method (DSM) and multiple impregnation method (IM) for CO_2_ methanation. The Ni@MIL-101(DSM) catalyst with Ni loading of 20 wt % exhibited surprisingly higher activity for CO_2_ methanation than Ni@MIL-101(IM), giving a CH_4_ turnover frequency (TOF) value of 1.63 × 10^−3^·s^−1^ at 300 °C. The author contributed the higher activity of Ni@MIL-101(DSM) to the more exposed Ni(111) facet, which was demonstrated by the result of DFT calculations that the Ni(111) plane has lower potential energy barrier (10.0 kcal/mol) for CO_2_ dissociation into CO_ads_ and O_ads_ than Ni(200) facet (20.3 kcal/mol).

The utilization of solar energy for the conversion of CO_2_ into valuable products is one of the best solutions to reduce carbon emission. Thus far, a range of photocatalysts, including TiO_2_, CdS, Zn_2_GeO_4_, graphite-like carbon nitride (g-C_3_N_4_), and other compounds have been successfully combined with MOFs to photocatalytically reduce CO_2_ [58,59,60,61,62,63,64,65]. For example, a metal-free semiconductor-composite (g-C_3_N_4_-ZIF-8) by growing ZIF-8 on the surface of g-C_3_N_4_ nanotubes for photocatalytic CO_2_ conversion into CH_3_OH was recently reported [63]. The ZIF-8 on the surface increases CO_2_ capture capacity, but impairs the surface charge transfer within the photocatalytic system due to the weaker electrical conductivity. The optimized ZIF-8 modified tubular g-C_3_N_4_ photocatalysts show the superior catalytic performance, giving a >3-fold yield of CH_3_OH, relative to the bulk g-C_3_N_4_.

**Figure 3:**
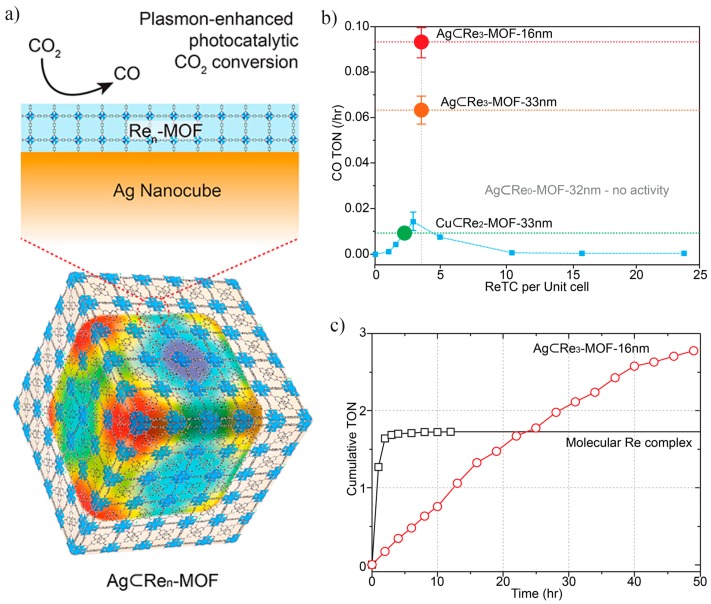
(**a**) Structure of Re*_n_*-MOF coated on Ag nanocube for enhanced photocatalytic conversion of CO_2_; (**b**) Photocatalytic CO_2_-to-CO conversion activity of Re*_n_*-MOFs (blue line), Ag⊂Re_0_-MOF, Cu⊂Re_2_-MOF, and Ag⊂Re_3_-MOFs with MOF thickness of 16 and 33 nm; and, (**c**) Stable performance of Ag⊂Re_3_-MOF when compared to molecular H_2_ReTC. Reproduced with permission from Reference [64]. Copyright 2016, American Chemical Society.

The separation efficiency of the photoinduced charge carriers plays an important role in photocatalysis [18,66]. The introduction of metal atoms into MOF photocatalysts may suppress the recombination of photoinduced electrons and holes and significantly increase their photocatalytic activity. Yaghi et al. [64] reported the construction of Ag⊂Re*_n_*-MOF with enhanced photocatalytic activity for CO_2_ reduction to form CO, which resulted from the cooperation of the spatially confined photoactive Re centers and the intensified near-surface electric fields at the surface of Ag nanocubes (Figure 3a). A fine balance of proximity between photoactive centers is needed for cooperatively enhanced photocatalytic activity in Re*_n_*-MOFs. The optimal Re_3_-MOF structure with the highest turnover on silver nanocubes shows a 7-fold enhancement in CO evolution rate over Re_3_-MOF under visible light (Figure 3b). Furthermore, Ag⊂Re_3_-MOF structure exhibits long-term stability of up to 48 h when compared to molecular H_2_ReTC, and the CO produced from Ag⊂Re_3_-MOF almost doubles from that of H_2_ReTC after 48 h (Figure 3c).

**Figure 4:**
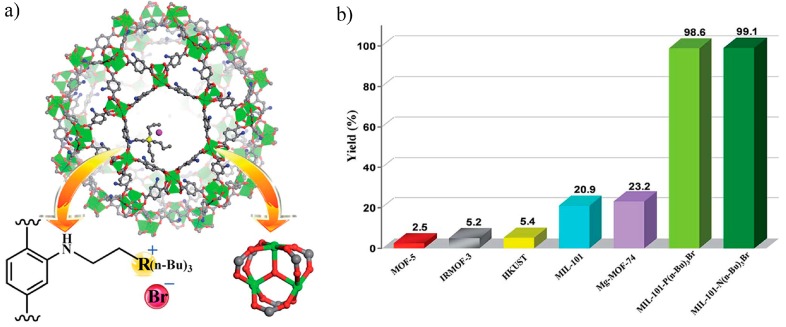
(**a**) The structure of the catalysts MIL-101-N(*n*-Bu)_3_Br and MIL-101-P(*n*-Bu)_3_Br (Cr: green; C: gray; O: red; N: blue; Br: amaranth, and R=N or P); (**b**) The yield of PC from the cycloaddition of PO and CO_2_ catalyzed by different MOF catalysts. Reproduced with permission from Reference [72]. Copyright 2015, The Royal Society of Chemistry.

Besides, catalytic processes that convert CO_2_ into cyclic carbonates have been widely investigated due to their high atom efficiency and high value products [67,68]. A growing number of MOFs have been employed as catalysts for the formation of cyclic organic carbonate. Meanwhile, a strategy for combining MOFs with functional species, like ILs, together to form heterogeneous catalysts has been developed to enhance the catalytic activity for conversion of CO_2_ into cyclic carbonates [69,70,71]. Shi and co-workers [72] reported two IL functionalized bifunctional catalyst, MIL-101-N(*n*-Bu)_3_Br and MIL-101-P(*n*-Bu)_3_Br, as prepared by the covalent post functionalization of MOFs (Figure 4a). Due to the synergy of two functional sites including Lewis acid sites in the MOF framework and nucleophilic anion in the ILs, the MIL-101-N(*n*-Bu)_3_Br, and MIL-101-P(*n*-Bu)_3_Br catalysts showed the highest yield to propylene carbonate (PC) (>98%) for the cycloaddition reaction of CO_2_ and propylene oxide (PO), when compared to other MOFs under mild and co-catalyst free conditions (Figure 4b). Similarly, the catalytic activity of ILs supported ZIF-90 for the PO-CO_2_ cycloaddition reaction is remarkably enhanced as compared to ZIF-90 [73].

### 3.3. Catalytic Hydrogen Production

#### 3.3.1. Catalytic Hydrogen Generation from Chemical Hydrides

A number of metal NPs that were immobilized in MOFs with high catalytic activities have been investigated for hydrogen generation from liquid chemical hydrides, such as aqueous formic acid (HCOOH), ammonia borane (NH_3_BH_3_), and hydrazine (N_2_H_4_). Due to the high specific surface area and tunable pore size, the loading of metal NPs inside MOFs could control the size of NPs in the confined cavities and produce monodispersed metal NPs, affording an enhanced catalytic performance.

**Figure 5:**
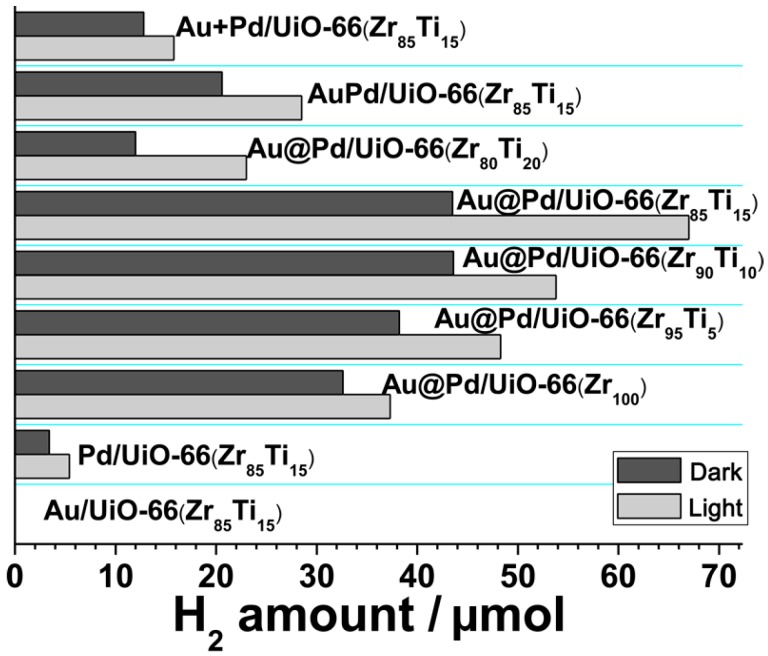
Amount of H_2_ produced through formic acid dehydrogenation in the dark (black bars) or under visible light irradiation (gray bars, λ > 420 nm, 320 mW·cm^−2^) over different catalysts. Reproduced with permission from Reference [91]. Copyright 2017, American Chemical Society.

In 2011, it was reported firstly that bimetallic Au-Pd NPs immobilized into MIL-101 and ethylenediamine (ED)-grafted MIL-101 (ED-MIL-101) were efficient catalysts for the hydrogen generation from formic acid at a mild temperature [74]. The obtained Au-Pd/ED-MIL-101 catalyst (Au-Pd loading: 20.4 wt %; Au:Pd = 2.46) exhibits superior catalytic performance when compared to those of other counterparts, giving a TOF of 106 h^−1^ at 90 °C. The enhanced activity is contributed to the smaller particle sizes of the Au-Pd alloy by the introduction of the electron-rich ED into MIL-101 and the strong synergistic effects between Au and Pd. This study reveals a new approach to immobilize metal NPs for hydrogen generation from formic acid. Since then, metal NPs, including monometallic NPs, bimetallic NPs, and even trimetallic NPs that were supported on various MOFs for hydrogen generation have been widely investigated [28,29,30,31,32,74,75,76,77,78,79,80,81,82,83,84,85,86,87,88,89,90,91]. Table 2 summarizes the catalytic activities of different catalysts for hydrogen production from HCOOH, NH_3_BH_3_, and hydrazine. Among these MOF-supported metal NPs catalyst, the Au_0.28_Pd_0.47_Co_0.25_/MIL-101-NH_2_ catalyst exhibits 100% selectivity to H_2_ and the drastically high activity for the dehydrogenation of formic acid at room temperature with a TOF value of 347 h^−1^ [86]. Interestingly, Yamashita and co-workers [91] recently reported the use of the synergistic catalysis of plasmonic Au@Pd NPs, and Ti-doped amine functionalized MOFs for enhancing hydrogen production from formic acid at room temperature. As shown in Figure 5, the activity of all supported Au@Pd catalysts is greatly enhanced under visible light irradiation, and the Au@Pd/UiO-66(Zr_85_Ti_15_) shows better catalytic performance under visible light irradiation when compared to those under dark conditions, giving a high TOF of 200 h^−1^. The similar cooperative promoting effect from both NPs and photoactive MOF was examined on a noble-metal-free catalyst Ni@MIL-101 for hydrogen generation from ammonia borane under assistance of visible light irradiation [88].

In order to reduce the deposition of the metal NPs on the outer surface of MOF support, Xu et al. [28] successfully developed a double solvent approach combined with hydrogen reduction to immobilize the ultrafine Pt NPs inside the pores of MIL-101. The resulting Pt@MIL-101 composite with 2 wt % Pt loading is highly active for H_2_ generation from aqueous ammonia borane at room temperature. By using a similar approach, AuNi [29], RuNi [30], AuCo [31], and CuCo [32] alloy NPs are also successfully encapsulated in the pores of MIL-101. The resulting MIL-101 supported bimetallic NPs catalysts present remarkably high catalytic activity for hydrolytic dehydrogenation of ammonia borane, giving the highest TOF of 66.2 min^−1^, 272.7 min^−1^, 23.5 min^−1^, and 19.2 min^−1^, respectively.

The hydrogen generation from the dehydrogenation of hydrous hydrazine has also been investigated using MOF-supported bimetallic NPs catalysts with high selectivity [76,78,80,84,85]. Highly dispersed bimetallic Ni-Pt NPs are successfully immobilized onto ZIF-8 via a facile liquid impregnation method, followed by co-reduction [76]. The activity of the composite catalysts strongly depended on the Ni-Pt composition, and the catalyst Ni_80_Pt_20_/ZIF-8 exhibits the highest activity at 50 °C with a 100% hydrogen selectivity and a TOF value of 90 h^−1^. Following a similar approach, Luo and his co-workers [78,80,84,85] successfully encapsulated the bimetallic Ni-Pt, Ni-Rh, and Ni–Ir NPs with different compositions into the cavities of ZIF-8 and MIL-101. The optimal catalysts, Ni_88_Pt_12_@MIL-101 [78], Ni_66_Rh_34_@ZIF-8 [80], Ni_42_Rh_58_@MIL-101 [84], and Ni_85_Ir_15_@MIL-101 [85], enable the rapid and complete decomposition of hydrazine in an aqueous alkaline solution, with a 100% H_2_ selectivity at 50 °C, giving the highest TOF value of 375.1 h^−1^, 140 h^−1^, 344 h^−1^, and 464 h^−1^, respectively.

#### 3.3.2. Catalytic Hydrogen Production from Water

**Figure 6:**
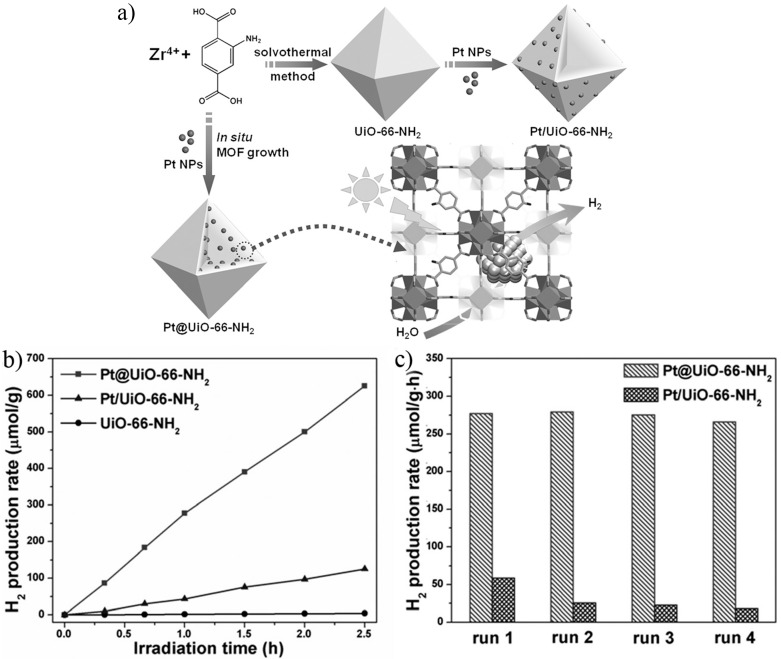
(**a**) Schematic illustration for the synthesis of Pt@UiO-66-NH_2_ and Pt/UiO-66-NH_2_; (**b**) The photocatalytic hydrogen-production rates of UiO-66-NH_2_, Pt@UiO-66-NH_2_, and Pt/UiO-66-NH_2_; and, (**c**) Recycling performance comparison between Pt@UiO-66-NH_2_ and Pt/UiO-66-NH_2_. Reproduced with permission from Reference [36]. Copyright 2016, John Wiley & Sons, Inc.

The photocatalytic water splitting is an ideal method for producing hydrogen. Developing new hybrid photocatalysts combined with MOFs is considered to improve the charge transfer/separation efficiency. Some researchers incorporate Pt NPs into MOFs to enhance hydrogen evolution reaction (HER) activity [36,92,93]. For example, approximately 3 nm Pt NPs are incorporated into or supported on UiO-66-NH_2_, to afford Pt@UiO-66-NH_2_ and Pt/UiO-66-NH_2_, respectively (Figure 6a) [36]. A high H_2_ evolution rate of 257.38 µmol·g^−1^·h^−1^, which is approximately 150 and 5 times higher than that of the parent MOF and the Pt/UiO-66-NH_2_, respectively, was achieved using a Pt@UiO-66-NH_2_ photocatalyst with the optimum Pt loading amount (2.87 wt %) (Figure 6b). In addition, as shown in Figure 6c, the Pt@UiO-66-NH_2_ shows better catalytic recyclability than that of Pt/UiO-66-NH_2_ because Pt@UiO-66-NH_2_ effectively restrains aggregation or leaching of Pt NPs during the reaction.

Recently, some functional components, including nickel particles [94], metal sulfides (e.g., MoxSy, NixSy, CdS) [95,96,97,98], reduced graphene oxide (rGO) [99], g-C_3_N_4_ [100], and POMs [101] have been employed to replace precious Pt and improve the photocatalytic activity for HER. Excellent photocatalytic activity was achieved as well. Interestingly, MoS_2_ increases the photoactivity for the HER and was superior to Pt as a co-catalyst [96]. 1 wt % MoS_2_/UiO-66-CdS obtained a high H_2_ evolution rate of 25,770 µmol·g^−1^·h^−1^, approximately 2-fold higher than that of 1 wt % Pt/UiO-66-CdS. Lin et al. [101] reported the successful encapsulation of tetra-nickel-containing Ni_4_P_2_ POMs into the pores of highly stable MOFs. The integration of the photosensitizing MOF framework and the POM catalyst allows for facile multi-electron transfer to enable an efficient HER, with turnover numbers as high as 1476.

### 3.4. Organic Reactions

#### 3.4.1. Oxidation of Alcohols and Hydrocarbons

MOF composites have been employed as efficient catalysts for the selective oxidation of alcohols, which are commonly considered as the central reactions in organic chemistry. Li et al. [102,103,104] investigated the catalytic performance of a series of MOF composites that were fabricated with different metal NPs (Au, Pd, and Pt) in liquid-phase aerobic oxidation of alcohols. The resulting Au/MIL-101, Pd/UIO-67, and Pt/DUT-5 composite catalysts exhibit excellent catalytic activities in a variety of alcohols oxidation reactions under base-free conditions, with exceeding 99% conversions and up to 100% selectivity to cinnamyl aldehyde. The authors attributed the superior catalytic activity to the beneficial synergetic effects of the electron donation and nano-confinement that is offered by the MOF framework. 

Other composite systems, including Au NPs, metal nanoclusters, POMs, CdS, and rGO, also exhibit high catalytic activities for selective oxidation of various alcohol substrates to aldehydes [105,106,107,108,109,110,111]. For example, Zhu et al. [106] recently reported the atomically precise nanoclusters@MIL-101 composites, prepared by using MOFs as the size-confining templates for the first time. Highly dispersed Au_13_Ag_12_@MOF composites exhibited favorable catalytic activity in the oxidation of benzyl alcohol toward benzaldehyde, exceeding 75% conversion and 100% selectivity.

Besides, the aerobic oxidation of hydrocarbons catalyzed by MOF based nanohybrid catalysts has also been explored. Various active species, including Au, AuPd, PtPd alloy NPs, POMs, and grapheme oxide (GO), combined with several MOFs were proved to be active and selective in the oxidation hydrocarbons with molecular oxygen [108,112,113,114,115]. For example, the AuPd/MIL-101 catalyst exhibited a superior activity and selectivity in the oxidation of cyclohexane to cyclohexanone and cyclohexanol (KA-oil) when compared with those of their pure metal counterparts and an Au+Pd physical mixture, which may be correlated to the synergistic alloying effect of bimetallic AuPd NPs [113]. Cyclohexane conversion exceeding 40% is achieved (TOF = 19,000 h^−1^) with >80% selectivity to KA-oil under mild solvent-free conditions. Recently, H_3+*x*_PMo_12−*x*_V*_x_*O_40_@MIL-100 (Fe) (*x* = 0, 1, 2) hybrids were prepared by the encapsulation of POMs within a metal–organic framework using a hydrothermal method [116]. The hybrids show greatly improved catalytic performance for the allylic oxidation of cyclohexene using H_2_O_2_ as green oxidant. In particular, the hybrid H_4_PMo_11_VO_40_@MIL-100 (Fe) leads to 85% cyclohexene conversion, 91% selectivity for 2-cyclohexene-1-one under optimized conditions, as well as excellent stability and reusability.

#### 3.4.2. Hydrogenation Reaction

Hydrogenation is a key reaction that is extensively employed in industry. The Pd, Pt, Ru, and Ni NPs, and their bimetallic NPs immobilized by MOFs have been proven to be active catalysts in the hydrogenation of a wide range of substrates including alkenes [117,118,119,120], alkynes [121,122], aromatics [123], nitro-aromatics [124,125], ketones [126], aldehydes [127,128], and other compounds [129,130].

**Figure 7:**
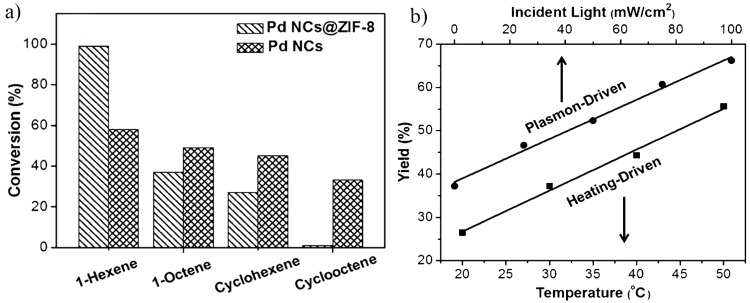
(**a**) Conversions of the hydrogenation of various alkenes over Pd NCs@ZIF-8 and Pd NCs under 100 mWcm^−2^ full-spectrum irradiation at room temperature; (**b**) The yields of the hydrogenation of 1-hexene with 1atm H_2_ over Pd NCs@ZIF-8 under full-spectrum irradiation with different light intensities at room temperature or upon heating at different temperatures. Reproduced with permission from Reference [24]. Copyright 2016, John Wiley & Sons, Inc.

When considering the molecular sieving capability of the pore apertures of MOFs, the encapsulated metal NPs in the internal frameworks of MOFs have been demonstrated to exhibit interesting size selectivity for the hydrogenation of different alkenes [25,131,132]. For example, Jiang et al. [24] reported the beneficial combination of the photothermal effects of metal nanocrystals with the favorable properties of MOFs for efficient and selective catalysis. As shown in Figure 7a, the Pd nanocubes (NCs)@ZIF-8 composite shows significantly different catalytic activity for olefins with different molecular sizes. For *n*-hexene, approximately 100% conversion can be achieved, while large molecules, such as cyclooctene (5.5 Å), cannot access the ZIF-8 shell with a pore aperture of (3.4 Å), resulting in negligible conversion. More interestingly, the catalytic performance of the Pd NCs@ZIF-8 composite in the hydrogenation of *n*-hexene is significantly improved by light irradiation (Figure 7b).

**Figure 8:**
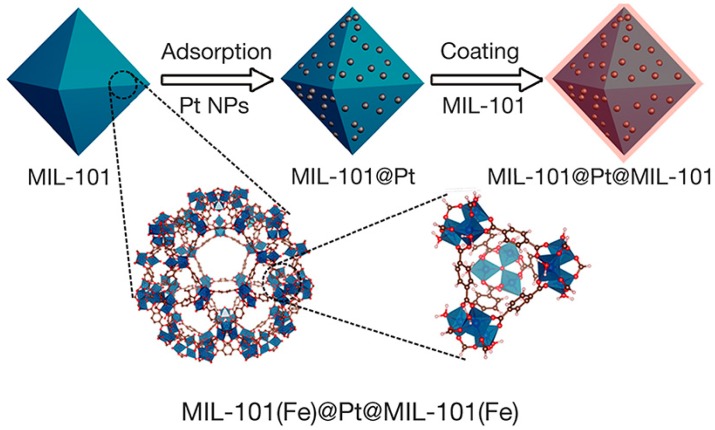
Synthetic route to generating sandwich MIL-101@Pt@MIL-101. Reproduced with permission from Reference [128]. Copyright 2016, Nature Publishing Group.

Recently, Zhao and co-workers [128] successfully fabricated a sandwich nanostructured catalyst with a layer of Pt NPs encapsulated between a core and a shell made of MIL-101 (Figure 8). The MIL-101(Cr)@Pt@MIL-101(Fe) catalyst, with a shell of thickness 2.9 nm, exhibits excellent selectivity (95.6%) and almost full conversion (99.8%) for the selective hydrogenation of cinnamaldehyde to cinnamyl alcohol. Furthermore, the shell thickness in sandwich nanostructures can be used to tune the activity and selectivity of sandwich-type catalysts. The design of a sandwich nanostructured catalyst provides a potential strategy for designing selective catalysts for many important, but highly challenging, reactions. 

#### 3.4.3. Catalytic C-C Coupling

C–C coupling reactions, including Suzuki-Miyaura, Ullmann, Heck, Sonogashira, and Stille reactions are among the most versatile and important reactions in organic synthesis [20]. Pd is the most common catalyst in promoting these coupling reactions, and hence MOF supported Pd NPs have been developed as C–C coupling catalysts [133,134,135,136]. Trzeciak and his co-workers [137] synthesized the supported Pd NPs on a nanoscale Ni-MOF by using a facile impregnation process with solvent in situ reduction method, which was confirmed as a highly active heterogeneous catalyst for the Suzuki-Miyaura coupling reaction between aryl halides and phenylboronic acid. This composite catalyst gives high yields of different coupling products with good recycle stability, indicating the excellent performance of the catalyst.

### 3.5. Catalytic Remediation of Pollutants

Wastewater that is produced in many industrial processes often contains toxic organic compounds and heavy metals, including organic dyes, phenols, hydrocarbons, pharmaceuticals, hexavalent chromium (Cr(VI)), and so on. It is necessary to reduce the concentration of pollutants before discharging the wastewater into the aquatic environment. Otherwise, various environmental and health issues could be induced. Among the physical, chemical, and biological technologies that are applied for the removal of pollutants, heterogeneous catalysis, including photocatalysis, has been demonstrated to be an efficient, economical, and green technique to degrade pollutants into easily biodegradable or less toxic compounds. Recently, MOF based composites with noble metal NPs, metal oxide/sulfides, GO, and other compounds have been proved to be a new class of catalysts, which are usable in the catalytic degradation of organic pollutants and Cr(VI) [138,139,140,141,142,143,144,145,146,147,148,149,150,151,152,153,154,155,156,157,158,159].

#### 3.5.1. Catalytic Degradation of Organic Pollutants

Advanced oxidation processes (AOPs) are increasingly adopted for the degradation of organic pollutants, owing to the in situ generation of highly reactive and nonselective radicals, such as ^•^OH, ^•^O_2_^−^, ^•^OOH, and ^•^SO_4_^−^ [160]. In AOPs, heterogeneous photocatalysis has been developed to be a green and efficient approach to degrade various organic pollutants. Since the amenability to design MOFs by controlling the constituent metal ions and organic linkers, MOFs-based photocatalysts with various morphology and structure have a great potential in the degradation of organic pollutants and environmental remediation.

Qiu et al. [154] firstly rationally fabricated a new type of core–shell Fe_3_O_4_@MIL-100(Fe) composite with a magnetic core and a designable MOF shell. This magnetic recyclable composite exhibits photocatalytic activity for methylene blue (MB) degradation under both UV-vis and visible light irradiation with good recycling stability. Based on a similar process, a yolk-shell Co_3_O_4_@MOF was successfully prepared via the fabrication of a uniform Fe-doped MOF-5 shell around Co_3_O_4_ NPs [150]. The degradation rate of 4-chlorophenol in the presence of peroxymonosulfate over 99% within 60 min was achieved by the Co_3_O_4_@MOF composite with satisfactory reusability.

Recently, Yuan et al. [142] reported a novel core–shell In_2_S_3_@MIL-125(Ti) photocatalytic adsorbent, which was prepared by a facile solvothermal method. The integrated composite exhibits excellent adsorption affinity, as well as superior photocatalytic activity, for the removal of tetracycline (TC). The photo-degradation efficiency for TC within a 60-min visible light irradiation is 63.3%, which is much higher than that of bare In_2_S_3_ or MIL-125(Ti), indicating the beneficial synergistic effect between MIL-125(Ti) and In_2_S_3_. The core–shell composites also reveal good performance for the removal of TC from various real wastewaters. Similarly, the CdS/MIL-53(Fe) and CdTe QDs/Eu-MOF composites show high photocatalytic activity in the degradation of Rhodamine B (RhB) and Rhodamine 6G in water at room temperature under light irradiation [143,146]. 

Alternatively, the bismuth oxyhalides (BiO*X*, *X* = Cl, Br, I) and MOFs composites are also effective in improving photocatalytic performance. BiOBr/UIO-66, BiOBr/NH_2_-MIL125(Ti), BiOBr/CAU-17, and BiOI/MIL-88B(Fe) nanocomposites have been reported as highly efficient photocatalysts for the degradation of organic pollutants [147,148,149,158].

Unlike metal semiconductor photocatalysts, g-C_3_N_4_, a metal-free semiconductor, has been successfully employed as a semiconductor photocatalyst for pollutant degradation [141,144]. Yuan et al. [141] successfully combined g-C_3_N_4_ with MIL-125(Ti) by a facile solvothermal method. The resulting g-C_3_N_4_/MIL-125(Ti) composite catalyst exhibits an excellent catalytic performance with good reusability and stability. The optimal photocatalytic performance is obtained at the g-C_3_N_4_ content of 7 wt % for MIL-125(Ti), on which the rate of RhB photodegradation is 0.0624 min^−1^, which is about 2.1 and 24 times higher than that of pure g-C_3_N_4_ and MIL-125(Ti), respectively.

#### 3.5.2. Catalytic Cr(VI) Reduction

Catalytic reductive transformation of Cr(VI) to Cr(III) is a promising method to perform effective remediation of Cr(VI), because Cr(III) is much less toxic and can be easily precipitated and removed due to its lower solubility in water in contrast to highly water-soluble and toxic Cr(VI).

Xu, Yu and Trivedi’s groups reported active MOF-supported noble metal (Pt, Pd) NPs catalysts for the reduction of Cr(VI) to Cr(III) using formic acid [145,152,157]. The resulting composites exhibit favorable catalytic performance, as demonstrated by the short time of complete reduction of Cr(VI) into Cr(III). Importantly, Yu et al. [152] proposed a reasonable mechanism for the catalytic reduction of Cr(VI) into Cr(III), as evidenced by a series of experiments. Formic acid is first adsorbed on the surface of metal NPs, and is then is dehydrogenated to produce H_2_ and CO_2_, according to Equation (1). The generated hydrogen, with high reduction capacity, then reduces Cr(VI) into Cr(III), as shown in Equation (2):

HCOOH → H_2_ + CO_2_ (1)

Cr_2_O_7_^2−^ + 8H^+^ + 3H_2_ → 2Cr^3+^ + 7H_2_O (2)

**Figure 9:**
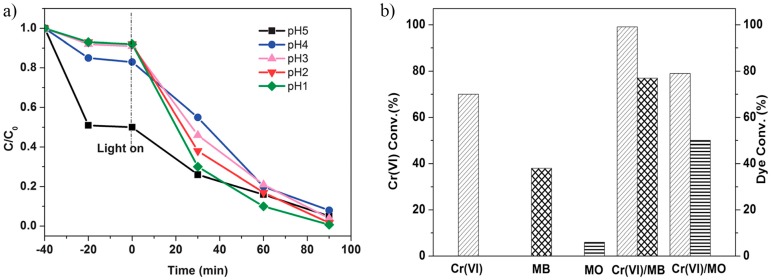
(**a**) Photocatalytic reduction of aqueous hexavalent chromium (Cr(VI)) with different amounts of H_2_SO_4_ solution (0.2 M); and (**b**) Simultaneous photocatalytic reduction of Cr(VI) and degradation of dyes (10 ppm) over Pd@UiO-66-NH_2_ under visible light irradiation (λ ≥ 420 nm). Reproduced with permission from Reference [155]. Copyright 2013, The Royal Society of Chemistry.

Wu et al. [155] firstly reported the simultaneous degradation of different categories of pollutants, such as Cr(VI) and organic dyes, by using MOF-supported Pd NPs as a dual functional photocatalyst. As shown in Figure 9a, Pd@UiO-66-NH_2_ is highly active to almost completely reduce Cr(VI) into Cr(III) within 90 min under visible light irradiation (λ ≥ 420 nm) at a pH range of 1–5, which is ascribed to the intimate interfacial contact between Pd and UiO-66-NH_2_, leading to the efficient charge separation. More interestingly, the reduction of Cr(VI) is visibly enhanced when the organic dye (MB or methyl orange (MO)) is added into the reaction system. In the binary systems of Cr(VI)/MO and Cr(VI)/MB, the reduction ratios of Cr(VI) are 79% and ~100%, respectively, which are obviously higher than that in the single system (70%) (Figure 9b). Meanwhile, the presence of Cr(VI) also promotes the photocatalytic degradation of MB and MO, demonstrating the beneficial synergic effect between the photocatalytic reduction and oxidation process. Wu et al. [138] also reported that the MIL-53(Fe)-rGO composite exhibits high photocatalytic activity in simultaneous oxidation of organic dyes and the reduction of Cr(VI).

Moreover, the rGO-UiO-66-NH_2_ and rGO-MIL-53(Fe) nanocomposites were successfully assembled by electrostatic attractive force for surface attachment [138,159]. The optimal photocatalytic performance is obtained at the rGO content of 2% and 0.5% for UiO-66-NH_2_ and MIL-53(Fe), for which the reduction ratio of Cr(VI) is even up to 100% under visible light illumination within 100 min and 80 min, respectively.

Recently, Wang et al. [139] employed ZnO colloidal spheres as template and zinc source to successfully fabricate core-shell ZnO@ZIF-8 composite. The resulting composite exhibits an enhanced selective photocatalytic reduction of Cr(VI) between Cr(VI) and MB, which is attributed to selective adsorption and permeation effect of the ZIF-8 shell.

## 4. Conclusions and Outlook

MOFs have been considered as ideal catalysts with clearly defined and designable crystal structures, high surface areas, tunable and uniform pore structures, and special confined nanopore microenvironments [27]. Some intrinsic weaknesses (like the lack in the catalytically active sites and limited thermal and chemical stability), however, hinder them from implementing the full potential for catalyst uses. With the improvement of the MOF syntheses, MOFs with enhanced thermal and chemical stability have been more and more available [9,14,27,35]. To enhance the catalytic properties of MOFs, MOF based metal and metal oxide NP composites have been prepared and successfully applied for many reactions. The ‘‘ship in bottle’’ strategy, ‘‘bottle around ship’’ strategy, and one-step synthesis strategy have been exploited for the syntheses of such composites. Among these strategies, the ‘‘ship in bottle’’ approach has some limitations in the precise control of the size, morphology, and composition of incorporated NPs. On the contrary, the size, morphology, and composition of NPs can be better controlled with the ‘‘bottle around ship’’ approach. The one-step approach is straightforward, but it usually needs the functional groups in organic linkers or solvents to trap the NPs precursors and stabilize the NPs that are formed.

The present studies have demonstrated that the MOF based NP composite catalysts exhibit excellent catalytic performances for CO oxidation, CO_2_ conversion, hydrogen production, organic reaction, and pollutant degradation. The high catalytic performances are contributed to the beneficial synergistic effects of MOFs and active sites of NPs. Significantly increasing applications of MOF based NP composite catalysts can be expected.

However, MOF composites, as heterogeneous catalysts, are still under its early developing stage. In the view of structural features, MOF composites consist of active species, metal ions and organic ligands, which are the potential factors influencing the catalytic performance. The relationship between the catalytic behavior and the structural features still needs to be investigated further. Further improvement in the thermal and chemical stability of MOFs is required. The present syntheses of MOFs are not cheap. Some syntheses are complex and time consuming, with difficulty in the scaling up. Innovation in the syntheses of MOFs is one of the future developments. Besides the improvement in the syntheses of MOFs for catalyst uses, the following studies are expected to further lead the progresses in the MOF composite catalysts:

(1) A principal advantage of MOFs is their designable structure with clear chemistry. When metal or metal oxide NPs are introduced, not clear chemistry may be caused because of not only various complex issues of NPs (like defects), but the interaction between MOFs and NPs. This is the most significant challenge for the future development with opportunities. In order to develop a size, structure, and location controllable preparation of NP/MOF composites, the interaction between metal or metal oxide and the MOF support needs to be further investigated. The functionalization and modification of MOFs needs to be also further investigated. The involvement of organic links in the catalytic reaction and the formation of intrinsic structure of MOFs with NPs generate many fundamental thermodynamic and kinetic issues for catalyst investigations. The reported studies are mostly limited within the experimental exploitation of MOFs for various reactions. Regarding the difficulty in the catalyst characterization, more theoretical studies are needed [45,161,162].
(2) For the composites by the pre-synthesized NPs, the polymer capping agent, like PVP, has some negative effects on the catalytic properties. Alternatives to such agents should be investigated. In this regard, biomolecules, like peptide, can be a nice candidate [163,164,165]. Especially, the peptide metal composite can serve as an excellent nitrogen dopant, which will be good for the improvement of the electronic properties of the catalysts, especially, for photocatalyst and electro-photocatalyst.
(3) Since MOFs are now mostly thermal sensitive materials, innovation in the post treatment or modification of the NP/MOF composites at low temperatures is extremely important. We attempted to use the room temperature electron reduction [166,167] for the preparation of noble metal/MOF composite catalyst. The present result shows the NPs cannot disperse well into the pores of MOFs. Further improvement is required. Other new approaches are also needed.
(4) An important future direction is the preparation of NP/MOF composites, together with gels, graphene, porous polymers, ionic liquids, and others, especially for the blooming photocatalytic and photo-electro-catalytic applications [168,169].
(5) The development of MOF based solid acid and solid base will create more opportunities for NP/MOF composite catalysts [170].
(6) MOF itself has been demonstrated to be an excellent single site catalyst, with which metal nodes in MOFs mimic homogeneous catalysts not only functionally but also mechanistically [171,172]. It provides a blueprint for the development of advanced heterogeneous catalysts with similar degrees of tunability to their homogeneous counterparts. It could open new ways for the investigations of NP/MOF catalysts or MOF supported NP catalysts, which possess characteristics and advantages of both heterogeneous and homogeneous catalysts.

## Figures and Tables

**Table 1:** Catalytic activities for CO oxidations on MOF-supported nanoparticles (NPs) catalysts.

Active Species (wt %)	MOF	*T*_50_^a^ (°C)	*T*_100_^b^ (°C)	Reference
Au (5%)	ZIF-8	170	210	[41]
Au (4%)	UIO-66	155	225	[43]
Pt (5%)	NH_2_-MIL-101(Al)	170	207	[42]
Pt (5%)	MIL-101(Cr)	118	150	[28]
Pt (5%)	*N*-UiO-67	100	120	[45]
Ag (5%)	Cu_3_(BTC)_2_	100	120	[48]
Pd (2.7%)	MIL-53(Al)	100	115	[44]
Pd (2.9%)	MIL-101(Cr)	92	107	[47]
Pd (5%)	Ce-MOF	77	96	[46]
Co_3_O_4_	ZIF-8	58	80	[49]

^a^ Temperature for 50% conversion of CO into CO_2_; ^b^ Temperature for 100% conversion of CO into CO_2_.

**Table 2:** Catalytic activities of different catalysts for hydrogen generation from chemical hydrides.

Catalyst	Chemical Hydrides	T (°C)	TOF (h^−1^)	Refernence
AuPd@ED-MIL-101	HCOOH	90	106	[74]
Ag_20_Pd_80_@MIL-101	HCOOH	80	848	[79]
Ag_18_Pd_82_@ZIF-8	HCOOH	80	580	[82]
Au_28_Pd_47_Co_25_/MIL-101–NH_2_	HCOOH	25	347	[86]
Ag_25_Pd_75_@NH_2_-UIO-66	HCOOH	25	103	[87]
Au@Pd/NH_2_-UiO-66(Zr_85_Ti_15_)	HCOOH	30	200	[91]
Ni@ZIF-8	NH_3_BH_3_	25	504	[75]
Ru@MIL-101	NH_3_BH_3_	25	10680	[77]
Ni@MIL-101	NH_3_BH_3_	25	3238	[88]
Pd@Co@MIL-101	NH_3_BH_3_	30	3060	[81]
Au_7_Ni_93_ @MIL-101	NH_3_BH_3_	25	3972	[29]
Ru_30_Ni_70_@MIL-101	NH_3_BH_3_	25	16363	[30]
Au_6_Co_94_@MIL-101	NH_3_BH_3_	25	1410	[31]
Cu_30_Co_70_@MIL-101	NH_3_BH_3_	25	1176	[32]
CuCo@MIL-101	NH_3_BH_3_	25	3102	[90]
FeCo@MIL-101	NH_3_BH_3_	25	3048	[90]
NiCo@MIL-101	NH_3_BH_3_	25	2658	[90]
Ni_80_Pt_20_@ZIF-8	Hydrazine	50	90	[76]
Ni_66_Rh_34_@ZIF-8	Hydrazine	50	140	[80]
Ni_88_Pt_12_@MIL-101	Hydrazine	50	375	[78]
Ni_42_Rh_58_@MIL-101	Hydrazine	50	344	[84]
Ni_85_Ir_15_@MIL-101	Hydrazine	50	464	[85]
